# A medical student perspective on question banks as a learning resource: a prescription for passing?

**DOI:** 10.1186/s12967-024-05811-6

**Published:** 2024-11-26

**Authors:** Siddhant Kansal, Rhea Elise Patel

**Affiliations:** Imperial College School of Medicine, Exhibition Road, London, SW7 2AZ UK

Dear editor,

Question banks (QBs) provide medical students with personalised and convenient learning, underpinning their widespread popularity [1]. The pitfalls, however, are often overlooked, and it is unwise to excessively rely on QBs as a primary learning method as explored in the article. We present our experiences with QBs and offer some suggestions on how they could be introduced or used more effectively by students.

The article proposes the idea of supporting students in creating their own questions to address the shortage of medical-school specific questions. However, based on our experience in tutorials as well as informal group revision sessions, we find that students lack the experience and knowledge required to write multiple choice questions with an appropriate level of complexity. A more effective approach might be to offer teaching on crafting exam-style questions, enabling students to contribute to a university-specific QB as part of their learning tutorials [2].

We have observed, however, that creating a university-specific QB promotes learning purely for exam purposes instead of cultivating a strong command of concepts to ensure clinical competence. This approach might conflict with the General Medical Council’s introduction of the Medical Licensing Assessment, which aims to reduce variations in syllabi and promote a standardised knowledge base for all graduates [3]. Further to this, it is also important to consider whether these QBs would remain up to date with evolving curricula and diverse teaching methods.

Notably, the COVID-19 pandemic compelled students to adopt more independent styles of learning, often relying on external online resources, such as QBs [4]. From personal experience, students in our year became more accustomed to pattern recognition instead of abstract thinking as a learning strategy. This shift may mean that earlier-year medical students approach learning differently to their senior peers.

As a field of study, medicine is particularly susceptible to rapid development. It is clear, the increasingly discursive nature of medical education has rendered rote learning inadequate. The growing emphasis on the biopsychosocial foundations of clinical practice calls for a more dynamic appreciation of clinical knowledge [5], as supported by widespread curricular reforms. At Imperial College London, a reformed course was launched in the 2019-20 academic year to align with the rapidly advancing NHS and anticipate its future. Similar reforms nationwide suggest that independent QBs may not reflect the unique and transforming medical school curricula.

Considering the vast amount of knowledge required and the individualised nature of revision, it is understandable that we as students place different subjective levels of importance on various revision methods, as their approaches to studying are highly personalised. The value we as students attribute to each resource is determined by their particular role it serves. QBs may be used to consolidate material, incrementally assess progress, or serve as a primary tool for learning exam-specific content. Our perceived effectiveness of these resources is directly determined by their utility. Students adjust their revision priorities throughout the academic year; conducting interviews with students at different timepoints would provide deeper insights into their evolving views on the use of QBs.

We are hopeful that QBs will advance with developments in the medical field, ensuring their ongoing relevance and effectiveness.

## Data Availability

Not applicable due to nature as a Letter to the Editor, all available online publicly through hyperlinks in references stated.

## Abbreviations

QBs: Question banks
